# Giant Abdominal Lipoma in Adult

**DOI:** 10.1155/2021/6610533

**Published:** 2021-03-02

**Authors:** Nihal Cinar Ozcan, Akay Edizsoy, Tahsin Colak

**Affiliations:** Department of General Surgery, Medical Faculty, Mersin University, 33110 Mersin, Turkey

## Abstract

Lipomas arising from the omentum are extremely rare in adults. Omental lipomas are typically asymptomatic, but very large ones may cause nonspecific abdominal symptoms and discomfort. Rarely they can cause omental torsion and present with an acute abdomen. We report a 41-year-old female patient with a giant lipoma (40 × 26 × 8 cm and 11,520 g) who presented with mild abdominal discomfort. Workup included abdominal ultrasound (USG) and magnetic resonance imaging (MRI). Surgical resection was performed without complication. No recurrence was observed during 4-year follow-up.

## 1. Introduction

Although lipomas are the most common encapsulated benign mesenchymal tumors of soft tissue, omental lipomas are extremely rare. We found no case series published in the global literature. Only a few case reports have been reported in the literature, and most of these are in childhood [1]. Furthermore, giant omental lipomas in adults are extremely rare. Less than 10 cases have been published so far in the literature [2–10]. Most of the cases were asymptomatic and diagnosed incidentally [2]. Some cases presenting with acute abdomen due to torsion or compression to other organs were reported [11, 12]. This report presents a patient with a giant lipoma which occupied nearly the entire abdominal cavity yet caused minimal ambiguous nonspecific symptoms and discomfort.

## 2. Case Presentation

**Figure 1:**
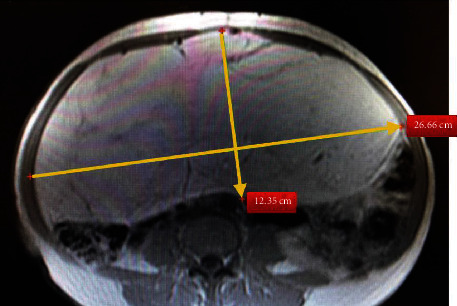
Axial view of magnetic resonance imaging shows that the lesion is encapsulated and lobulated.

**Figure 2:**
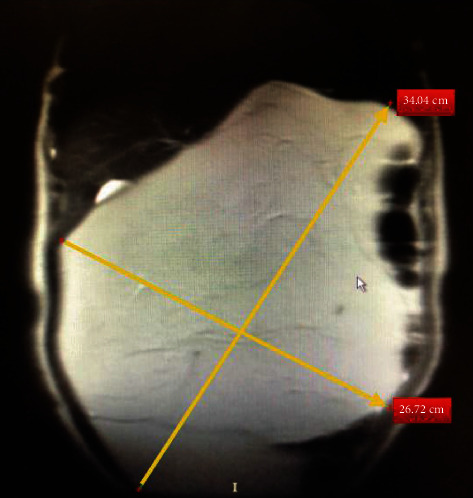
The lesion measured as approximately 34 cm × 26 cm in size on coronal view of magnetic resonance imaging.

**Figure 3:**
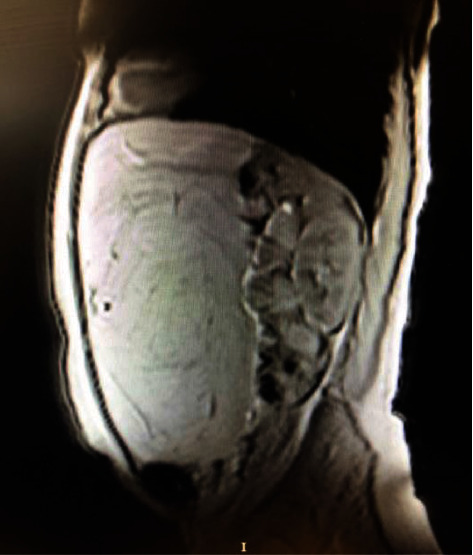
Sagittal view of magnetic resonance imaging shows that the lesion caused the intestines to move towards the posterior.

A 41-year-old woman was admitted to the hospital with mild abdominal pain, discomfort, and bloating on the abdomen. In recent years, the patient had mildly suffered from these symptoms. The patient did not complain of weight loss, nausea, vomiting, weakness, or anorexia, but she was worried about increasing waist circumference and some dyspeptic symptoms. Physical examination revealed a big, relatively mobile, nontender, smooth solid mass occupying almost the entire abdomen. There was no evidence of mechanical bowel obstruction. There was no history of any abnormalities of the digestive system, liver or renal failure, gynecological malignancy, or any other reason to cause abdominal ascites. All hematological and biochemical parameters were within normal range. Also, tumor markers (please be specific) were normal. A solid, well-defined mass lesion measuring approximately 40 cm in greatest diameter was reported on ultrasound (USG). On magnetic resonance imaging (MRI), T1-weighted axial and coronal MR images showed a hyperintense giant mass filling most of the abdomen and displacing the bowels. The lesion was also compressing the liver. In fat-saturated T1-weighted MR images, the signal of the mass was suppressed similarly to fat. The mass did not enhance with contrast. The lesion measured approximately 34 cm × 26 cm in size (Figures 1–3). Given the symptomatic nature of the mass, surgical resection was recommended. A laparotomy was performed by midline abdominal incision. Operative exploration revealed a giant mass with a smooth surface, encapsulated and lobulated that was originating from the greater omentum.

**Figure 4:**
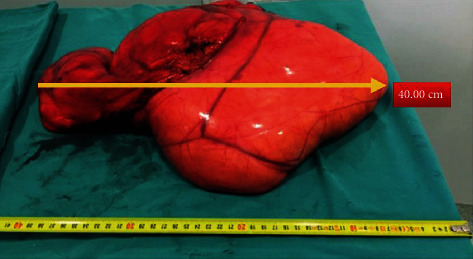
It was reported that the long diameter of the pathology specimen was measured as 40 cm.

It was extending along the falciform ligament to the posterior liver and filled the entire abdomen and pelvis caudad without strong adhesion to adjoining tissues and organs. The mass displaced the intestines posteriorly but did not cause obstruction. Also, the entire colon was intact. The mass was completely extracted without any violation of lipoma capsule. The final specimen measured 40 × 26 × 8 cm and weighed 11,520 g (Figure 4). Macroscopically, the specimen was a soft, encapsulated yellow fatty mass, and the cut surface consisted of homogenous fatty tissue. Pathological examination with hematoxylin-eosin staining revealed fine-encapsulated mature adipose tissue without atypia or malignant degeneration or necrotic tissue, consistent with a mature benign lipoma. In postoperative follow-up, liquid food was tolerated on the first postoperative day and solid food on the second day. She was discharged without any complications on postoperative day 3. No recurrence was observed during the 4-year follow-up.

## 3. Discussion

Although lipomas are the most common mesenchymal tumors on any part of the body, intra-abdominal lipomas are rarely seen, with omental lipomas being exceedingly rare [8]. Only about 20 cases that considered all ages have been reported [3], and less than 10 cases arising from the greater omentum in adults have been reported. A case was published as the largest ever reported omental lipoma weighing 6900 g in an adult [3]. The present case is one of the biggest omental lipomas reported with 11,520 g in weight. Early satiety, intermittent vomiting, abdominal discomfort due to displacement of intestines, and pressure effect of surrounding structures were frequently reported as the symptoms in the huge lipoma cases. Also, gradual abdominal distention and/or expansion in waist circumference were often seen in noncomplicated giant lipomas. The presented case had abdominal distention mildly, waist expansion without weight gain, and mild dyspeptic symptoms caused minimal discomfort. As in this case, most of the patients with omental lipoma were diagnosed after symptoms of abdominal discomfort were reported, or incidentally when the patients presented for different reasons [2].

USG and abdominal computerized tomography (CT) are the preferred imaging studies for abdominal lipomas. On USG, the lipomas appear as homogenous echogenic well-bounded masses. Because USG is largely user-dependent, most surgeons need further diagnostic imaging study including CT or MRI. CT shows the specific characteristics of fat content and aids in distinguishing from the other intra-abdominal masses including leiomyosarcoma, liposarcoma, fibroma, fibrosarcoma, or hemangiopericytoma [8]. CT also determines the size of the tumor and its relationship to neighboring structures [13]. It has also been reported that abdominal CT is useful for understanding the arterial supply of the tumor and its relationship with other organs [1]. MRI also provides excellent tissue characterization, with lipomas appearing identical to subcutaneous fat on all pulse sequences and any fibrous septa within exhibiting low signal intensity on T1- and T2-weighted images. The fatty nature of the tissue can be confirmed on chemical shift imaging and frequency selective fat suppression techniques [13]. In T1- and T2-weighted MR images, homogeneous low signal intensity should be detected. In distinction to liposarcoma, which may show thick, irregular, or nodular septations larger than 2 millimeters in both CT and MRI, fat-free nodular foci increased septal contrast [14]. In the current case, MRI was used to distinguish soft tissues, and the Doppler USG was used for vascularity. No findings suggesting other lesions with predominantly macroscopic fat include myelolipoma, angiomyolipoma, liposarcoma, teratoma, epiploic appendagitis, fat infarction, and mesenteric panniculitis were detected. Pereira et al. reported that MRI imaging techniques were often powerful tools in the noninvasive evaluation of these lesions. Knowledge of clinical, anatomic, and imaging features is important in formulating an appropriate differential diagnosis and guiding patient care, often obviating invasive diagnostic procedures [13].

In this patient, the lipoma was arising from the greater omentum and associated with the falciform ligament. It caused minimal discomfort and abdominal distention. But different unexpected clinical scenarios or acute abdominal syndrome occurred in some of the other cases. Hishiki et al. reported a case arising from the lesser omentum, presenting as an acute abdomen due to torsion [11]. Other reports include patients with omental lipomas in an inguinal hernia sac were noted [12, 15]. An omental lipoma case that caused amenorrhea and abdominal distention was reported; therefore, it was considered pregnancy [10]. Another patient who presented with abdominal pain developed an infarct in the lipoma originating from the falciform ligament [16]. The lipoma was excised from this patient who was operated on urgently due to the acute abdomen. A case with chronic diseases and bleeding spontaneously and disrupting the patient's hemodynamic was observed [17]. Transarterial embolization was applied to this patient, who was inoperable. In the presentation of large abdominal masses, well-differentiated liposarcomas should be included in the differential diagnosis, and reasonable effort should be made to discriminate these masses before the operation. Liposarcomas generally occur in the gluteal region, but they can rarely occur in the abdomen with similar characteristics of lipomas in other locations, such as an encapsulated and smooth surface without invasion of neighboring organs. Elevated serum amylase levels have also been reported [18].

Surgery is the treatment of choice for omental lipomas. Because lipomas are encapsulated and seldom infiltrate the surrounding structures, the excision technique is generally uncomplicated. However, every effort should be made for complete excision with an intact capsule to avoid recurrence. The rate of recurrence after excision is reported to be less than 5% [19]. Preoperative imaging is important for surgical planning. In the light of the literature, omental lipomas may create nonspecific signs or symptoms or be asymptomatic and may rarely lead to emergent surgery. Also, omental lipomas are surgical entities that need to be made a differential diagnosis from malignancies and can reach large sizes.

## 4. Conclusion

This case report revealed that omental lipomas can remain silent until they reach a giant size. In these cases, surgery is the definitive treatment option, and preoperative cross-sectional imaging is critical for surgical planning.
